# Relationships between structural stigma, societal stigma, and minority stress among gender minority people

**DOI:** 10.1038/s41598-024-85013-8

**Published:** 2025-01-23

**Authors:** Kristen D. Clark, Mitchell R. Lunn, Jae M. Sevelius, Carol Dawson-Rose, Sandra J. Weiss, Torsten B. Neilands, Micah E. Lubensky, Juno Obedin-Maliver, Annesa Flentje

**Affiliations:** 1 Department of Medical Sciences, Psychiatry, Uppsala University, Uppsala, Sweden; 2 The PRIDE Study/PRIDEnet, Stanford University School of Medicine, Stanford, CA USA; 3 Division of Nephrology, Department of Medicine, Stanford University School of Medicine, Stanford, CA USA; 4 Department of Epidemiology and Population Health, Stanford University School of Medicine, Stanford, CA USA; 5 Center for AIDS Prevention Studies, Department of Medicine, University of California, San Francisco, CA USA; 6 Department of Psychiatry, Columbia University Irving Medical Center, New York, NY 10032 USA; 7 Department of Community Health Systems, School of Nursing, University of California San Francisco, San Francisco, CA USA; 8 Department of Community Health Systems, UCSF Depression Center, University of California, San Francisco, CA USA; 9 Division of Prevention Science, University of California,, San Francisco, CA USA; 10 Department of Obstetrics and Gynecology, Stanford University School of Medicine, Stanford, CA USA; 11 Alliance Health Project, Department of Psychiatry, School of Medicine, University of California, San Francisco, CA USA

**Keywords:** Gender minority, Transgender, Minority stress, Structural stigma, Social attitudes, Policy, Stigma, Human behaviour, Risk factors

## Abstract

Structural stigma towards gender minority (GM; people whose current gender does not align with sex assigned at birth) people is an important contributor to minority stress (i.e., stress experienced due to one’s marginalized GM identity), although existing variables are unclear in their inclusion of social norms, or societal stigma, as a key component of the construct. We examined potential variables representing structural stigma, including variables that are inclusive of societal stigma, to identify those that most strongly relate to minority stress outcomes. We tested variables identified in the literature as measures of structural stigma inclusive of societal stigma (LGBT + Business Climate Index, state voting behaviors, and Google Trends search data), the most commonly used structural stigma variable (State Policy Environment Tally), and proxy variables (region, population density) for comparison. The relationships between structural stigma and minority stress model outcomes were tested in a sample of GM participants from The Population Research in Identity and Disparities for Equality (PRIDE) Study (*N* = 2,094) 2019 Annual Questionnaire using a structural equation model (SEM). Lower structural stigma (i.e., higher LGBT Business Climate Index) was associated with lower experienced stigma (β= -0.260, *p* < .01) and lower anticipated stigma (β= -0.433, *p* < .001). Greater conservative voting behavior was associated with less experienced stigma (β= -0.103, *p* < .01). Living in a more densely populated county was also associated with lower anticipated stigma (β=-0.108, *p* < .001) and greater identity outness (β = 0.053, *p* < .05). Two of the identified structural stigma variables that were inclusive of societal stigma (i.e., LGBT + Business Climate Index, conservative voting behaviors) and one proxy variable (population density) were associated with minority stress outcomes. However, the most commonly used variable for structural stigma (State Policy Environment Tally) was not associated with any outcomes. The State LGBT + Business Climate Index showed the most promise for use as a structural stigma variable in future research. The application of this variable should be investigated further to explore its association with health outcomes and to inform efforts to reduce health equity barriers experienced by GM people through addressing structural stigma in a manner inclusive of societal stigma.

**Supplementary Information:**

The online version contains supplementary material available at 10.1038/s41598-024-85013-8.

## Introduction

Broadly, stigma has been defined as the stereotyping, “othering,” and marginalization of a group of people in a manner that results in restricted access to resources, reduced social standing, and internalization of negative views^1^. Stigma toward gender minority (GM; people whose current gender identity does not align with commonly associated characteristics of the sex they were assigned at birth) people has been studied for many years and has been found to impact the social, emotional, and physical well-being of this group^2–4^, as well as many other marginalized populations^5–8^. Stigma has been operationalized into three primary constructs: individual stigma, interpersonal stigma, and structural stigma, (^1^; see defined terms in Table 1). Individual stigma is defined as the stigma processes within one’s self, such as the internalization of negative beliefs, attitudes, and shame about one’s marginalized identity^2^, or the act of concealing one’s identity and avoidance of interactions that could increase the risk of mistreatment^9^. Herek describes these types of stigma similarly but distinguishes felt stigma as a distinct construct apart from internalized stigma^9^. In this context, felt stigma refers to behavior changes, such as avoidance of healthcare or concealing marginalized identity, that may occur without direct experiences interpersonal stigma (termed enacted stigma by Herek). While other typologies stigma describe these behavior changes as individual stigma^1,3,10^, Herek points to the mechanistic function of felt stigma in driving these behaviors^9^. Interpersonal stigma is defined as experiences of stigma in interactions with others, such as discrimination, victimization, mistreatment, and rejection because of one’s marginalized status^2,9^. This aligns with Herek’s conceptualization of enacted stigma, which emphasizes direct acts of prejudice or discrimination, such as overt exclusion or mistreatment in interpersonal relationships^9^.

**Table 1:** Stigma and minority stress key terms.

Stigma Term	Definition	Source	Related Minority Stress Term	Definition	Source	Representation in Structural Equation Model
Stigma	Othering, marginalization of a group of people in a manner that results in restricted access to resources, reduced social standing, and the internalization of negative views	Link & Phelan, 2001	Minority Stress	Sources of chronic stress related to one’s sexual orientation or gender identity that impact the mental and physical health of that marginalized group.	Meyer, 2003; Testa et al., 2017	
Structural Stigma	Laws, policies, cultural norms, and other social-level factors that influence the social, economic, and personal wellbeing of a marginalized group	Hatzenbuehler et al., 2010, 2024	Not a part of the minority stress model	-	-	1. State Policy Environment Tally (most common measurement approach)
Societal Stigma	Degree to which society disapproves of a marginalized population, a subtype of structural stigma commonly unmeasured in current research approaches	Hasenbush et al., 2014	Not a part of the minority stress model	-	-	Includes direct measurement of societal stigma within the broader structural stigma construct: 2. State LGBT + Business Climate Index 3. Percentage of a state that voted Republican in 2020 election 4. Google Trends search for “transgender” by state Potential proxy, indirect measurement: 5. U.S. region 6. Population density
Interpersonal Stigma	Experiences of stigma such as discrimination, victimization, mistreatment, and rejection because one’s marginalized status. Also, described as enacted stigma.	White Hughto et al., 2015, King et al., 2021, Herek, 2007	Distal Stressors	Enacted or experienced stigmas, or the external experiences due to a minoritized sexual orientation or gender identity. Examples include discrimination, victimization, mistreatment, and rejection.	Meyer, 2003; Testa et al., 2017	Experienced Stigma- measured by 10 different types of discrimination or victimization with yes/no response options
Individual/Internalized Stigma	Internalization of negative beliefs, attitudes, and shame about one’s marginalized identity. Concealment of one’s marginalized identity and avoidance are also expressions, but differentiated in Herek’s typology as a separate construct.	White Hughto et al., 2015, King et al., 2021	Proximal Stressors	Internal processes, such as the negative self-view of one’s sexual orientation or gender identity or internalization of harmful societal views, or the concealment (or lack of outness) of one’s minoritized identity	Meyer, 2003; Testa et al., 2017	Anticipated Stigma-measured by 4 variables representing community safety and acceptance past and present Internalized Stigma- measured by the adapted Internalized Homophobia Scale-Revised Outness-measured by the adapted Nebraska Outness Scale
Felt Stigma	Differentiated from individual/internalized stigma by Herek, this term describes the experience of stigma without the requirement of a direct experience as a precedent, driving behavior to reduce risk. This term includes behaviors such as concealment and avoidance.	Herek, 2007	Proximal Stressors	See above.	Meyer, 2003; Testa et al., 2017	See above.

Structural stigma is defined as social norms, laws, and policies that influence the social, economic, and personal well-being of marginalized groups^10,11^. Herek (2007) highlights how structural systems perform the role of legitimizing stigma, perpetuating enacted and felt stigma by institutionalizing power imbalances and upholding social norms that marginalize minority groups^9^. Forms of structural stigma include laws that disproportionately target or penalize a group of people, or laws that, by omission, deny a marginalized group protection that is offered to others. One example of this would be the exclusion of gender identity from the list of protected groups included under hate crime laws^12,13^.

However, the construct of structural stigma has most commonly been operationalized in the extant literature as the laws and policies that influence the social, economic, and personal well-being of GM people^3,14^. Studies have often measured structural stigma using self-report items by asking GM participants to indicate their experiences of structural discrimination, such as employment, housing, or healthcare^3,15^. Yet self-reported items such as these are frequently enacted on an interpersonal level. Although they may have structural effects, they do not necessarily reflect a structural level enactment of stigma itself. A growing number of studies directly assess the laws, policies, or other social structures^4,16-22^ that directly impose structural forms of stigma. Studies examining these aspects of structural stigma are primarily conducted in the US and use a single measure, the State Policy Environment Tallies developed by the Movement Advancement Project^16,17,21^. Some studies have examined the measurement of laws and policies in the European Union at the country level to measure structural stigma toward LGBTQ + people, showing that discriminatory laws and policies were associated with lower satisfaction with life, high internalized stigma, and increased healthcare avoidance^18,23^. However, measurement of societal stigma, or social level attitudes or norms, is absent from these approaches.

Societal stigma is defined in this study as the social norms or societal disapproval of a marginalized group and is a subcomponent of structural stigma^11,19^. As was described within structural stigma, common measures of structural stigma include items querying GM people’s self-reported experiences of discrimination related to housing, employment, and healthcare, but these experiences are likely predicated by negative views of GM people held by the offending individual(s) who enacted harm^3,15^. However, that behavior may be emboldened, or even encouraged, by a lack of consequences for such behavior and a perceived sharing of negative views in the community^24,25^. Subsequently, self-reported forms of structural stigma are the consequences of both structural and societal factors and not always a direct measurement of structural stigma itself. Voting behavior in a US state is also an example of how structural stigma can be measured inclusive of societal stigma. Voting behavior is distinct, although related, to the policies enacted themselves^26^. Conservative voting behavior, i.e., the proportion of people in a US state who voted Republican in a major election, is associated with reduced healthcare access among GM people^26,27^. However, the use of conservative voting behavior as a measurement of structural stigma inclusive of societal stigma still has limitations. Voters may prioritize any number of issues that are not specific to GM people, and thus, it may not be directly representative of their attitudes toward this marginalized group specifically^28–31^. There is also the tendency for urban areas within a conservative state or country to often hold more favorable attitudes to GM people than other parts of the state, which is difficult to capture^32,33^. This can result in a disconnect between the laws and policies and the social norms or societal disapproval experienced by GM people more directly.

Geographical factors have been evaluated as a proxy for structural stigma toward GM people, such as the region or population density where one lives^26,34^. The region where one lives, particularly certain regions of the southern US, is associated with experiences of discrimination and victimization among GM people^15^ and fewer legal protections^11,20^. Similarly, residing in an area where there is lower population density has been associated with greater transphobic and homophobic attitudes^35,36^ as well as less access to housing and bathrooms that align with one’s gender or that are gender neutral^37^. This indicates that geographical measurement could represent the attitudes in an area or other unmeasured characteristics of structural stigma as there is evidence of its association with both the laws/policies aspect of structural stigma and experienced stigma, although it is not a direct measure of either construct in their entirety.

Stigma is an important consideration in the health and healthcare access among GM people, a relationship described by the minority stress model to explain health inequalities observed between GM people and the general population. The minority stress model describes how stigma related to one’s marginalized identity forms chronic stressors^38–41^. This source of stigma, in addition to the everyday stress that is experienced by the general population, results in poorer mental and physical health^38,39,41–45^. The minority stress model divides stressors into two main categories, distal and proximal stressors^41^. Distal stressors are enacted or experienced stigma, or external experiences related to their sexual and/or gender minority (SGM, commonly referred to as lesbian, gay, bisexual, transgender, or queer) identity. This is similar to how Link & Phelan describe these external experiences as interpersonal stigma^1,2^. Examples of distal stressors include physical violence, denial of services, identity rejection, or loss of employment^38,39^. The minority stress model shows that distal forms of stress (i.e., experienced stigma) may increase proximal forms of stress.

Proximal forms of minority stress are described as internal processes, such as the negative self-view of one’s GM identity or internalization of harmful views as well as the act of concealing one’s GM identity^39,41^. Proximal stressors have also been associated with poor mental and physical health outcomes. For example, anticipated stigma is associated with poor physical health and greater healthcare avoidance^16,17^. Concealment of one’s identity can be both a coping mechanism to experienced or anticipated stigma^44^ and a stressor associated with poor mental health outcomes among GM people^46^. Proximal stressors are described by Link & Phelan’s operationalization of stigma as individual stigma^1^. Although there is considerable overlap between Link & Phelan’s conceptualization of stigma and the minority stress model, structural stigma is absent from the latter’s conceptualization of stigma as a distinct source of chronic stress. This may be because structural stigma, including its aspect of societal stigma, may underly and contribute to the environments where minority stress takes place, driving health inequalities observed among GM people when compared to the general population. However, different approaches to measuring structural stigma should be evaluated for their fit with the minority stress model and compared. Therefore, the purpose of this study is to examine variables commonly used to measure structural stigma (i.e., State Level Policy Environments), variables of structural stigma inclusive of societal stigma (i.e., State LGBT + Business Climate Index, conservative voting behavior, Google Trends searches), and proxy variables for structural stigma (US region, and population density) to identify which is most strongly associated with outcomes from the minority stress model (i.e., distal stressors, defined as experienced stigma; and proximal stressors, defined as anticipated stigma, internalized stigma, and outness).

## Methods

We hypothesize that:

H1: Increased structural stigma will be associated with greater experienced stigma, anticipated stigma, internalized stigma, and less outness.

H2: Structural stigma variables inclusive of societal stigma (State LGBT + Business Climate Index, Google Trends, and Conservative voting behavior) will be more strongly associated with minority stress outcomes than commonly used variables (State Policy Environment Tally) or proxy structural stigma variables (US region, population density).

Data for the present study were collected using The Population Research in Identity and Disparities for Equality (PRIDE) Study’s 2019 Annual Questionnaire. The PRIDE Study is a national longitudinal cohort study of SGM people who reside in the US. The PRIDE Study is a community-engaged research study that acts in an ongoing partnership with an active Participant Advisory Committee via a community-engaged structure to catalyze SGM health research called PRIDEnet^47^. This committee reviewed and helped to inform the adaptations of measures used in The PRIDE Study to be inclusive of SGM communities and reviewed the study described herein. Please see Lunn et al. for a detailed description of The PRIDE Study^48^.

### Sample

Recruitment for The PRIDE Study included the involvement of PRIDEnet partners, which is a national group of SGM individuals and community organizations who assist in the recruitment of participants. Further recruitment efforts included online postings and distributions (e.g., blog posts, newsletters, advertising on social media), in-person outreach through conferences and SGM-related events, the distribution of The PRIDE Study promotional items, and word-of-mouth. Eligible participants included individuals who were 18 years and older, resided in the United States or its territories, identified as a sexual and/or gender minority person, were able to read and respond in English^48^, , and completed the annual questionnaire measures outlined in these analyses from June 2019 to May 2020 (*N* = 5,855). Participants who endorsed a GM identity (i.e., transgender woman, transgender man, non-binary, or “another gender identity”) were retained for analysis in this study (*N* = 2,226).

### Measures

#### Demographics

Demographic data collected included age, race/ethnicity, sexual orientation, gender identity, individual income, and highest level of education completed. State of residence was determined through participant-provided ZIP code. Age was calculated by subtracting a participant’s birth date, which was obtained upon study enrollment, from the date that the survey was begun. Race/ethnicity was measured with a categorical variable (select all that apply) with 8 options, including “None of these fully describes me.” Participants were asked, “If you had to choose only one of the following terms, which best describes your current sexual orientation?” Participants could then indicate asexual/demisexual/gray-ace, bisexual/pansexual, gay/lesbian, queer, straight/heterosexual, or “another sexual orientation.” Participants were provided a categorical variable that asked participants “If you had to choose only one of the following terms, which best describes your current gender identity?” Participants could then indicate cisgender man, cisgender woman, non-binary, transgender man, transgender woman, or “another gender identity.” This second variable was used to identify GM participants for the current study and to describe our sample in Table 2. Sex assigned at birth was measured with an item that asked, “What was your sex assigned at birth, for example on your original birth certificate?” Participants could choose either female or male. The highest level of education was measured by an ordinal variable with 10 options ranging from “no schooling” to “Professional degree,” coded this in our analyses as a 4-level variable (i.e., “no high school diploma,” “high school/GED graduate or some college,” “college degree [2- or 4-year],” and “graduate degree”). Individual income was measured by an ordinal 11-level variable ranging from $0 to $100,000, used in the analysis in these increments (collapsed in Table 2).

**Table 2:** Characteristics of the PRIDE study 2020 Annual Questionnaire participants (*N* = 2,094).

Variable	
*Personal characteristics*	Mean ± SD
Age, in years	31.6 (11.7)
Race/ethnicity^a^	n (%)
American Indian or Alaska Native	7 (0.3)
Asian	39 (1.9)
Black, African American, or African	29 (1.4)
Hispanic, Latino, or Spanish	56 (2.7)
Middle Eastern or North African	6 (0.3)
Native Hawaiian/Pacific Islander	2 (0.1)
White	1,895 (91.5)
Another race/ethnicity than is listed	38 (1.8)
Gender Identity	
Another gender identity	135 (6.5)
Non-binary	1,005 (4839)
Transgender man	653 (31.4)
Transgender woman	290 (13.9)
Sexual Orientation^a^	
Another Sexual Orientation	20 (1.0)
Asexual/Demisexual/Gray-Ace	289 (13.9)
Bisexual/Pansexual	644 (30.9)
Gay/Lesbian	351 (16.9)
Queer	692 (33.2)
Straight/Heterosexual Only	87 (4.2)
*Socioeconomic position*	
Annual individual income	
<$20K	1,019 (48.9)
$20K to <$40K	412 (19.8)
$40K to <$60K	257 (12.3)
$60K to <$80k	110 (5.3)
*≥*$80K	285 (13.7)
Level of Education	
No high school diploma	16 (0.8)
High school/GED graduate or some college	716 (35.9)
College degree (2- or 4-year)	762 (38.2)
Graduate degree	500 (25.1)

The number of participants in the study group with available data are reported as (*n*) and percent (%) of *n* for each variable.

^a^Category is not mutually exclusive; therefore, percentages may be greater than 100%.

Standard deviation = SD.

#### Structural stigma

Structural stigma variables were included in the analysis based on three categories. These variables were identified through extant GM health literature and reports from SGM non-profit organizations. The first category represents variables measuring structural stigma in a manner that includes some representation of societal stigma (i.e., not solely laws and policies but also a form of societal attitudes). These variables were included in subsequent analyses (i.e., State LGBT + Business Climate Index, conservative voting behavior, and Google Trends). The second category included the most commonly used measure of structural stigma in the existing literature, the Movement Advancement Project’s State Policy Environment Tallies, for comparison to the variables in the first category^3,49,50^. Lastly, the third category of structural stigma variables included those that may proxy structural stigma inclusive of societal stigma but do not represent a direct measurement (i.e., US region, population density). These variables were included because they capture geographical and cultural dimensions broadly. If this category of variables is more strongly associated with minority stress outcomes when compared to the variables in categories one and two, this may allude to aspects of structural stigma unmeasured by the other variables in the model. Subsequently, six variables were included in the final analysis.

##### State LGBT + business climate index

Out Leadership, an organization aimed at increasing SGM representation within corporations and other organizations, reports an annual index on how SGM-inclusive each state is to inform business leaders, organizations, and policymakers of “the costs created by policies that create minority stress”^51^. The LGBT + Business Climate Index incorporates data from multiple sources to represent both legal and societal stigma aspects of structural stigma. Sources include the Movement Advancement Project, the United States Transgender Survey, The Williams Institute, the Bureau of Labor Statistics, and the United States Treasury to create a single score for each state that ranges from 25 to 100. These points are derived from the evaluation of a state based on five domains. The “Legal and Nondiscrimination Protections” domain includes state laws related to sexual orientation and gender identity. The “Youth and Family Support” domain includes how supportive a state was toward SGM individuals starting a family by evaluating laws related to adoption, family leave, and how many SGM people in the state had children. This domain also measured state protections and resources for SGM children. The “Political and Religious Attitudes” domain includes whether a state has religious exemption laws and whether anti-SGM attitudes are present in political speeches, platforms, and actions on SGM-related laws by the state’s governor and senators. The “Health Access and Safety” domain includes accessibility of healthcare based on whether state health insurance includes gender-affirming interventions and whether private insurance is included under antidiscrimination laws. This domain also includes a reflection of safety by whether hate crime laws expressly include SGM people and whether HIV exposure or transmission is a criminal offense. The “Work Environment and Employment” domain measures workplace discrimination among SGM people, the rate of SGM people’s employment, the amount of SGM people earning less than USD 24,000 per year, and the comparison of food insecurity rates between SGM and non-SGM people in a state. Full scoring of each domain by state can be found at Out Leadership’s website^51^.

##### Conservative voting behavior

The number of votes for the Republican presidential nominee in the 2020 election^26,52^ was calculated as a percent of total votes cast in each state and included as a single continuous variable where higher percentages indicate greater structural stigma inclusive of societal stigma toward GM people^26,53–55^.

##### Google trends

Google Trends is a tool to determine the popularity of topics/search terms based on the number of search queries^56^. As Google is the most used search engine in the US, search results are particularly salient^57^. Previous literature has applied the search for a particular term to indicate the popularity of that subject and shown its association with state policies^58^, health information seeking^59^, and gender-affirming interventions^60,61^. This description of the popularity of the search term in a state could indicate greater comfort with GM identities and communities. However, its exploration in this context is novel and exploratory. We entered the term “transgender” and exported its popularity ranking by state in the US from June 2019 to May 2020 to align with our participant response window. These values provided a range from 0 to 100. These values were included as a single continuous variable with higher counts indicating the greatest proportion of total searches among the 50 United States.

##### State policy environment tally

The Movement Advancement Project reports each state’s SGM-inclusive legislation to create a state-level policy environment score^50^. For example, a state with employment anti-discrimination laws that explicitly apply to both sexual minority (i.e., people who identify as lesbian, gay, bisexual, and other diverse sexual orientations) and GM people would receive 2 out of 2 points, whereas a state without employment anti-discrimination laws for either sexual minority or GM people received 0 out of 2 points. If a state were to have no laws against SGM discrimination and *only* harmful policies (e.g., laws banning cities from passing anti-discrimination laws, or religious exemption laws) the state could have a negative total score. While our study is intended to evaluate structural stigma’s relationship with minority stress among GM people, we included policies related to sexual orientation in addition to gender identity. This was because the study sample is comprised entirely of GM people, but 96% also identified as sexual minorities. Subsequently, these policies are relevant to the lived experiences of GM people and previous research has supported this as sexual orientation-related policies have been associated with healthcare utilization, experienced stigma, and other outcomes among gender minority people^16,20,62^. The possible scores for each state range from − 17.5 to 35. These scores were extracted on October 8, 2019, as a single continuous variable with higher scores indicating more protections.

##### US region

Residing in the US South has been applied as a measurement of structural stigma and is associated with poorer healthcare access and greater interpersonal stigma reported among GM people^63,64^. A dichotomous variable was created indicating whether each participant resides in a state in the Southern US (1 = Southern US; 0 = not Southern US) based on the US Census Regions^65^.

##### Population density

Population density, by measuring whether a participant resides in a rural location or a more urban location, has been used as a measurement of structural stigma to indicate societal stigma and structural factors related to rurality, such as fewer legal protections^35,36,66,67^. Participant ZIP code was converted to Rural-Urban Continuum Codes^68^ in 2020, which categorizes the population density where one resides. Due to the limited population density variability in the sample, these codes were recoded to a dichotomous variable indicating metropolitan (1 = participant resides in a designated metropolitan county) and non-metropolitan (0 = participant resides in an area that is not designated as a metropolitan county).

#### Minority stress

##### Experienced stigma

This outcome^69^ was measured by using 10 dichotomous variables that queried participants’ about experiences of discrimination and victimization based on items from the National HIV Behavioral Surveillance surveys^70^. Seven items focused on types of discrimination experienced during the past year (i.e., employment, education, healthcare, housing, receiving services, interacting with law enforcement, and verbal harassment). Three variables focused on types of victimization experienced during the past year (i.e., physical attack/harm, violence from a romantic partner, and unwanted sexual contact). Participants who indicated “yes” to an item could indicate the reason they believe they experienced each stigmatizing event with a select-all-that-apply variable (i.e., ability/disability status, age, body size/weight/shape, gender expression, gender identity, race/ethnicity, sexual orientation, or something else). Participants who endorsed that they perceived the event to occur due to gender identity and/or gender expression were coded as 1. Participants who indicated “no” to past-year discrimination/victimization or who indicated “yes” but gave a reason other than gender identity or gender expression for the stigmatizing event were coded as 0. The responses were summed to create a single continuous observed experienced stigma variable, with a possible range from 0 to 10. Higher scores indicate more experienced stigma.

##### Anticipated stigma

This outcome was measured by variables querying perceived community acceptance and perceived community safety. Community acceptance and safety for GM people were assessed via self-report for both where the participant currently resides and where they were raised using four items adapted from Heck et al.^71^. For example, the first item queries “Overall, how safe for *gender minority* people is the community in which you *currently live*?” by answering on a Likert-type scale ranging from 0 to 4 (0 indicating “extremely unsafe” to 4 indicating “extremely safe”). The second item queries “Overall, how accepting of *gender minority* people is the community in which you *currently live*?” by answering on a Likert-type scale ranging from 0 to 4 (0 indicating “extremely unaccepting” and 4 indicating “extremely accepting”). These same two items were asked again, but participants were asked to respond based on where they were raised. The variable responses were recoded as inverse (0 indicating “extremely safe” to indicating “extremely unsafe”) to be more intuitively interpreted (greater numbers indicating greater anticipated stigma). The responses were summed to create a single observed continuous anticipated stigma variable, with a possible range from 4 to 16. Higher scores indicate higher levels of anticipated stigma.

##### Internalized stigma

The Internalized Homophobia Scale (IHP-R)^72^ was adapted from its use for internalized stigma related to sexual orientation to be reflective of GM people’s internalized stigma (e.g., “I wish I weren’t genderqueer, transgender, or gender minority”). Responses were summed and divided by 5, resulting in a continuous observed variable with a possible range of 1 to 5 with higher scores indicating increased internalized stigma^17,20,72^.

##### Outness

Outness was measured using the Nebraska Outness Scale, which indicates the degree to which participants are open about their GM status or concealment^73^. The measure was developed based on sexual orientation and was adapted for GM people but has not yet been validated for this population. The measure included 10 items, including two subscales with 5 items that measured concealment and 5 items that measured disclosure of GM status. This measure was adapted for GM people by including gender identity in lieu of sexual orientation and providing examples specific to GM people’s experiences, such as “e.g., not correcting people when they use a name or pronoun that is not accurate for you.” A Likert-type scale for each item ranged from 1 to 5 (1 indicating “disagree strongly” to 5 indicating “agree strongly”). Each subscale was totaled and averaged to provide an observed variable with a single continuous score that ranged from 1 to 5, where higher scores indicated greater outness and lower scores indicated greater concealment.

#### Analysis

We evaluated missing data patterns among the variables of interest. A total of 132 participants had not responded to any of the minority stress outcome items and were dropped a priori from further analyses. The model was estimated using full information maximum likelihood, which is appropriate for handling complex survey data and accounting for any missing data remaining. Cronbach’s alpha (α) was used to determine internal consistency among minority stress outcomes, excluding experienced stigma as this is a formative measure^74,75^. An internal consistency was considered appropriate when α *≥* 0.7. Descriptive statistics to describe the characteristics of the sample and alpha coefficients for the minority stress variables were generated using STATA 15^76^. Structural equation modeling (SEM) was used to test the relationship between structural stigma and minority stress outcomes within our GM sample using M*plus* 8^77^. Minority stress outcomes were included in the model as bidirectional correlations as these data are cross-sectional and causality cannot be evaluated. In the broader literature, there is also little evidence to inform the direction of the relationship. For example, increased interpersonal stigma is associated with increased internalized stigma^16^, yet increased internalized stigma is also a predictor of increased interpersonal stigma^92^. Standard errors and test statistics were adjusted for participants’ state of residence using robust cluster-adjusted variance estimation. Variables were introduced into the model in two steps^78^: an unadjusted model identifying the relationships between minority stress outcomes (experiences of stigma, anticipated stigma, internalized stigma, and outness) developed based on the minority stress model^38,39,41^ and structural stigma variables, and lastly, an adjusted model incorporating covariates (i.e., age, race/ethnicity, sexual orientation, level of education) to account for their contribution to the variance and ensure that the estimates are as accurate as possible. These covariates are a key consideration in understanding broader social determinants of health, even though they are not the primary focus of this analysis^14,17,26,27^. Power analysis using the N: q rule estimated that 740–940 participants (depending on the final number of structural stigma variables) were needed^79^. We reported both unstandardized and standardized regression coefficients and associated test statistics for the model relationships.

## Results

A total of 2,094 GM participants were included in the final analyses (Table 2). Non-mutually exclusive descriptions of race/ethnicity showed that approximately 76.4% of participants were solely white, while 14.1% were white and another race or ethnicity. Approximately a third of participants identified as queer, or bisexual/pansexual (33.2% and 30.9%, respectively), almost 17% as gay or lesbian, 14% as asexual/demisexual/gray-ace, while 4.2% were heterosexual/straight. Approximately a third of the sample (34.9%) had a high school level education or less. Almost half of the sample (48.9%) reported an individual income of less than USD 20,000 per year. Cronbach’s alpha coefficients for outness and internalized stigma were acceptable (α = 0.70). Alpha coefficients and descriptive statistics for minority stress and structural stigma variables are presented in Table 3.

**Table 3:** Descriptive statistics for minority stress and societal stigma variables (*N* = 2,094).

Variable	α	*n* (%)	M (SD)	Range
*Minority Stress Outcomes*				
Experienced Stigma (Past-Year)				
Verbal Harassment		705 (31.7)		
Physically Attacked or Injured		55 (2.5)		
Physical Violence from Romantic or Sexual Partner		19 (0.9)		
Unfair Treatment at or While Applying for Work		296 (13.3)		
Unfair Treatment While Renting/Buying a Home or Unfair Eviction		59 (2.7)		
Received Poorer Service Than Others at Restaurants/Stores/Businesses/Agencies		372 (16.7)		
Unfair Treatment While a Student at School or Other Educational Setting		175 (7.9)		
Denied or Provided Lower Quality Medical Care		270 (12.1)		
Denied or Provided Lower Quality Mental Health Care		142 (6.4)		
Unfair Treatment or Harassment from Police or Law Enforcement		75 (3.4)		
Unwanted Sexual Contact		104 (4.7)		
Anticipated Stigma	0.73			
Perceived Safety Where Currently Live			1.8 (0.9)	0–4
Perceived Safety Where Raised			2.6 (1.0)	0–4
Perceived Acceptance Where Currently Live			1.7 (1.0)	0–4
Perceived Acceptance Where Raised			2.9 (1.0)	0–4
Internalized Stigma	0.72		1.9 (0.8)	1–5
Outness	0.82		4.6 (2.2)	0–10
*Structural Stigma*				
State-Level Policy Environments			18.0 (12.8)	-2.5-34
State LGBT + Business Climate Index			68.1 (19.0)	31.2–90
Conservative voting behavior			0.5 (0.1)	0.3–0.7
Google Trends for “transgender”			79.4 (7.7)	62–100
US Region (Living in the South)		571 (27.3)		
Population Density		1,871 (89.4)		

**Fig. 1:**
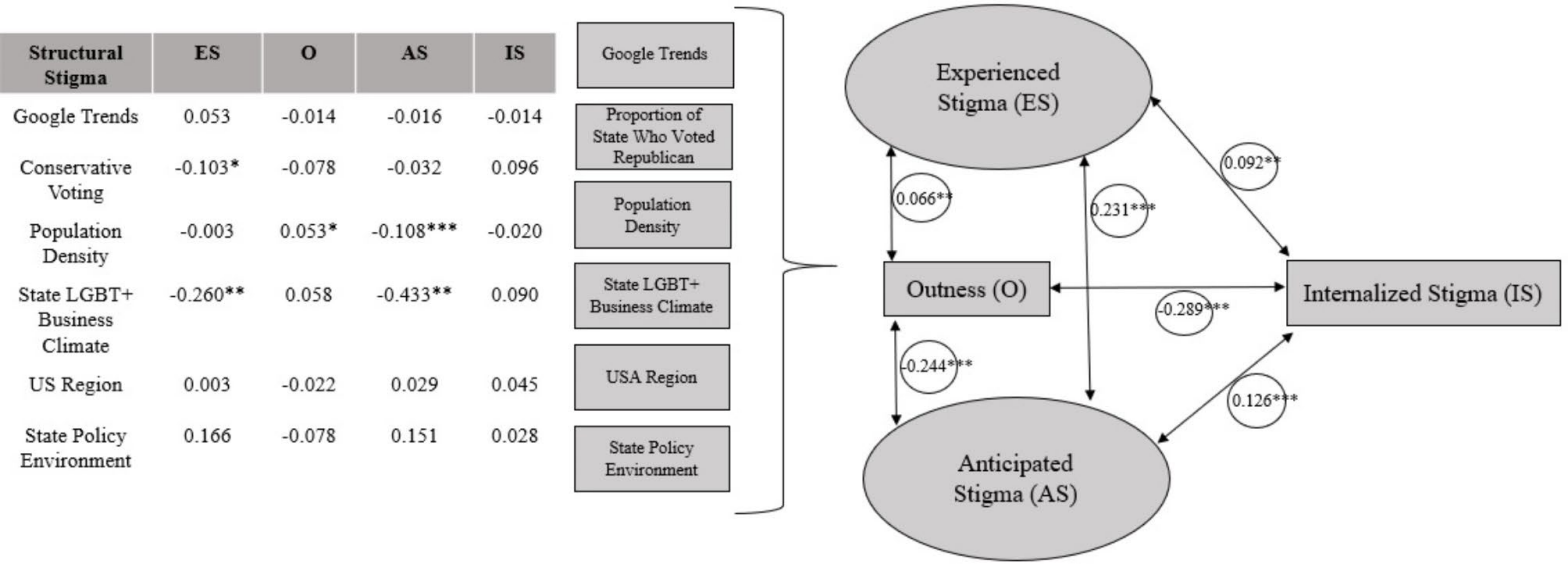
Structural model of societal stigma and its association with minority stress outcomes (*N* = 2,094). Standardized coefficients = β. *p* < .05*, *p* < .01**, *p* < .001***..

The unadjusted model, with estimated standardized path coefficients, is presented in Supplemental Table 1, with standard errors. In the adjusted model, each minority stress outcome adjusted for covariates (i.e., race/ethnicity, sexual orientation, gender identity, level of education, and age;Fig. 1). The adjust model showed that greater experienced stigma was associated with greater anticipated stigma (β = 0.231, *p* < .001) and greater internalized stigma (β = 0.092, *p* < .01). Greater anticipated stigma was associated with greater internalized stigma (β = 0.126, *p* < .001). Greater outness was associated with less internalized stigma (β = -0.289, *p* < .001), less anticipated stigma (β = -0.244, *p* > .001), but greater experienced stigma (β = 0.066, *p* < .01). When compared to the unadjusted model, the inclusion of covariates did not alter which variables were statistically significant in their association with minority stress outcomes. Effect sizes generally only decreased slightly, indicating that some of the variance in the outcomes was accounted for by the added covariates. The exception was the State LGBTQ+ Business Climate Index, whose association with anticipated stigma strengthened slightly (β = -0.375 to -0.433). These changes suggest that the covariates had limited influence on the primary predictors, with the State LGBTQ + Business Climate Index standing out as potentially more robust in its relationship to anticipated stigma when accounting for additional factors.

State LGBT + Business Climate Index was negatively associated with experienced stigma (β =-0.260, *p* < .01) and anticipated stigma (β = -0.433, *p* < .001); meaning that the more SGM-inclusive a state was, the lower the experienced and anticipated stigma were. However, it was not associated with internalized stigma (*p* = .499) or outness (*p* = .631). Population density was negatively associated with anticipated stigma (β = -0.108, *p <* .001) and positively associated with outness (β = 0.053, *p <* .05), meaning that GM participants who lived in more metropolitan areas reported less anticipated stigma and more outness. It was not associated with experienced stigma (*p* = .902) or internalized stigma (*p* = .436). Conservative voting behavior was negatively associated with experienced stigma (β = -0.103, *p* < .01), meaning GM participants who lived in a state that voted more conservatively reported less experienced stigma. It was not associated with any other minority stress outcomes. Google Trends, US region, and state-level policy environment were not associated with any minority stress outcomes. Estimates and standard errors appear in Table 4.

**Table 4:** Unstandardized estimates, standard errors, and standardized estimates for the structural equation model of minority stress and its association with structural stigma variables (adjusted model; *N* = 2,094).

Outcome Variable	Google Trend			State Policy Environment Tally			State LGBT + Business Climate Index			Conservative Voting Behavior			US Region			Population Density		
	B	SE	β	B	SE	β	B	SE	β	B	SE	β	B	SE	β	B	SE	β
Experienced Stigma	0.006	0.022	0.033	0.019	0.077	0.166	**-0.020**	**0.079**	**-0.260**	**-1.658**	**0.132**	**-0.103**	0.008	0.022	0.003	-0.014	0.024	-0.003
Anticipated Stigma	0.001	0.022	0.001	0.035	0.093	0.151	**-0.066**	**0.105**	**-0.433**	-1.038	0.083	-0.032	0.191	0.051	0.029	**-1.020**	**0.025**	**-0.108**
Internalized Stigma	-0.002	0.043	-0.015	0.002	0.096	0.028	0.004	0.133	0.090	0.891	0.069	0.096	0.085	0.045	0.045	-0.055	0.025	-0.020
Outness	0.004	0.022	0.013	-0.014	0.087	-0.078	0.007	0.120	0.058	-1.913	0.064	-0.078	-0.110	0.041	-0.022	**0.378**	**0.025**	**0.053**

Bolded values indicate *p* < .05*.

## Discussion

We hypothesized that states with higher structural stigma would be associated with greater experienced stigma, anticipated stigma, internalized stigma, and less outness. In the adjusted model, less structural stigma was associated with less experienced stigma, less anticipated stigma, and more outness, partially supporting our hypothesis. Yet internalized stigma was not associated with any of the structural stigma variables.

To compare potential structural stigma variables and the strength of their association with minority stres outcomes, we tested six variables within three categories based on their conceptual fit. One category included three variables that were inclusive of societal stigma (i.e., State LGBT + Business Climate Index, conservative voting behavior, Google Trends searches), one category with a single variable that was not inclusive of societal stigma but commonly used to represent structural stigma (i.e., State Policy Environment Tally), and the last category with two variables that proxy structural stigma inclusive of societal stigma but do not directly measure either (US region, and population density). None of the six variables representing structural stigma were associated with all minority stress outcomes.

We also hypothesized that structural stigma variables inclusive of societal stigma (State LGBT + Business Climate Index, Google Trends, and Conservative voting behavior) would be more strongly associated with minority stress outcomes than commonly used variables (State Policy Environment Tally) or proxy structural stigma variables (US region, population density). However, in partial alignment with our hypothesis, two of the variables inclusive of societal stigma, the State LGBT + Business Climate Index and conservative voting behavior, were associated with one minority stress outcome. One proxy variable, population density, was associated with two minority stress outcomes.

The State LGBT + Business Climate Index indicated that greater inclusivity in a state, or less structural stigma, was associated with less experienced stigma and less anticipated stigma. The State LGBT + Business Climate Index was unique in that its development involved the representation of multiple forms of structural stigma: SGM policy protections, economic indicators, and societal stigma in the form of SGM attitudes and statements from governors and senators in each US state^51^. The inclusion of economic indicators and societal stigma differentiated this variable from the State Policy Environment Tally, which was not associated with minority stress outcomes, despite the two variables’ shared use of SGM policy protections. This is likely due to societal stigma’s role as an underlying contributor to structural stigma. The attitudes of those in a community or state are at least partially reflected by the policies that are passed and whether those policies are enforced, a variable that accounts for multiple forms of structural stigma that could be most closely representative of societal stigma as a construct. Particularly in this case where societal stigma is measured based on political representatives public statements/speeches/platforms. Therefore, the State LGBT + Business Climate Index may be more closely representative of people’s experiences of structural stigma than State Policy Environment Tallies and could be the most appropriate variable for structural stigma measurement.

Population density, or whether one lives in a metropolitan environment as reflected in the present study, was associated with two minority stress outcomes among GM people: less anticipated stigma and greater outness. Living in more metropolitan environments may increase access to social support and community resources^66^, both of which indicate lower structural stigma and potentially more positive social attitudes (i.e., lower societal stigma). One consideration is that social support has been found to moderate the relationship between minority stressors and poor mental health outcomes and is more accessible in metropolitan environments^43,80^. Less metropolitan environments tend to be associated with more societal stigma, such as transphobic and homophobic attitudes^35,36,81^. This could be due to the lower visibility of GM people because of the need for concealment for personal safety, which may lead to a lack of resources for support^67,82^. Therefore, population density may be indicative of both structural and interpersonal stigma in a more localized geographical area than state-level variables.

Conservative voting behavior, represented in the present study as the percentage of a state that voted Republican in the 2020 election, was associated with less experienced stigma, although not with any other of the minority stress outcomes in our study. This is contrary to previous literature that found greater experiences of stigma in states with higher conservative voting behavior, such as refusal of general healthcare services^26^. This could be due to the composition of our sample, which was predominantly GM people residing in metropolitan areas, which tend to be more accepting or where community connections and resources are often situated^32,83^. While a state may have reported a higher portion of conservative votes, our participants who reside in those states may live in areas that did not vote majority conservative; for example, the state of Texas voted predominantly conservative but Travis County, Texas, which includes the progressive city of Austin, voted majority liberal in the preceding election^52,84^. The role of outness, or concealment, should also be considered. The decision to conceal one’s identity can be protective as hiding one’s identity may prevent one from mistreatment. This may be an explanation for the less experienced stigma with more conservative state voting behavior^85,86^. This finding could underline the importance of selecting the appropriate structural stigma variables that incorporate societal stigma into their measurement and address community level variability.

Higher Google Trends search results by state for the term “transgender” were not associated with minority stress outcomes. This variable communicates the frequency of a search relative to the state’s population but does not provide context to the searcher’s intent. For example, when states have highly publicized legislation related to GM people, searches may increase due to political dialogue and be unrelated to one’s attitude toward GM people. It is also likely that GM people themselves are contributing to searches in these states to gather information on the political environment and news, as a form of self-care, or to seek social support^87,88^. Previous literature has found an association between searches for racial slurs and mortality rates among Black Americans^89^; however, this is not an equal comparison since the use of a slur is not the same as the term “transgender.” The use of derogatory terms communicates an underlying attitude or intent, which makes the search frequency a clearer interpretation of attitudes. Future work should develop a new measure for structural stigma inclusive of societal stigma, evaluate its relationship as a contributor to experiences of minority stress, and to inform opportunities for intervention. Evaluation of societal stigma as a key component of structural stigma is needed because whether the broader, dominant society accepts or denies the existence and rights of a marginalized group influences the policies, resources, behaviors, and individual-level beliefs that follow. One opportunity could involve the exploration of the variables outlined in this study to determine if a new index could be developed to more closely predict experiences of minority stress. Our findings illustrate that the variables that emerged as most promising includes societal attitudes as part of structural stigma measurement. State LGBT + Business Climate Index incorporated policy, economic conditions, and societal attitudes in its determination of state scores^51^, indicating areas where structural stigma impacts the access to resources and means available to GM people. For example, structural stigma may mean that a state has less protection for GM people who may be denied or fired from employment due to their gender identity or expression (experienced stigma), which in turn impacts their earnings potential^90^. However, the attitudes reflected in this variable may indicate the likelihood that individuals in that state are to act on negative views of GM people. Population density was less clear in terms of its explicit relationship with structural stigma and underlying societal attitudes and could be representative of GM people’s ability to access resources and community support. Yet, its association with outness and anticipated stigma indicates a need to explore structural stigma further as a proxy for unmeasured characteristics, particularly for how they relate to outness which was not associated with any other structural stigma variables.

### Limitations

The present study is an important contribution to our understanding of current and future approaches to structural stigma measurement and its relationship with minority stress experienced by GM people. The findings are further strengthened by a lack of common method bias through its application of external structural stigma variables that are inclusive of societal stigma and the integration with self-report survey data^91^. However, it is important to note that limitations remain. Structural stigma, inclusive of societal attitudes, is one consideration for understanding the drivers of interpersonal and individual stigma described in the minority stress model and by Link & Phelan^1^. Other contributing factors – such as race/ethnicity and sexual orientation – were included in our model as covariates, but were not examined at these intersections; therefore, our findings are not fully generalizable to communities of color and other minoritized groups. However, when the unadjusted and adjusted model results were compared, the inclusion of these variables as covariates had little effect on the model. Yet it is important to note that the sample was almost 80% exclusively white, which limits the power to detect such differences. Similarly, our sample was over 50% non-binary GM people. This indicates that the experiences of structural stigma faced by transgender women and transgender men may be obscured. Our discrimination and victimization items allow for only a dichotomous participant response (yes/no); therefore, we are unable to determine the frequency, severity, or any other nuanced characteristics of these experiences. These findings were generated with cross-sectional data; therefore, we cannot infer causality. This has a high impact on the interpretation of the associations between minority stress variables. The bidirectional modeling of minority stress outcomes acknowledges that unmodeled factors may serve as common causes of these variables, contributing to unexplained variance within the model results. Despite these limitations, the bidirectional approach allowed us to capture the complex interrelationships among minority stress outcomes, which is valuable in the absence of longitudinal data.

It is also important to note that none of the variables representing structural stigma were developed explicitly for this purpose. As a result, no single variable is a perfect representation of structural stigma inclusive of societal stigma. US region and population density are proxies that may reflect aspects of structural stigma and societal stigma as a subcomponent but do not measure any specific part directly. Google Trends as a variable poses the additional limitation that multiple search terms cannot be combined. Therefore, other terms used to conduct internet searches related to GM people are not reflected in the variable and affect its accuracy. Additionally, none of the included structural stigma variables allowed for a more localized representation of participant experiences (e.g., within the community where a person lives, works, and interacts most frequently). Regarding the conceptual measurement of minority stress within the present study, the items used to represent experienced stigma, anticipated stigma, internalized stigma, and outness have not been validated for use in this context. However, previous analyses of minority stress among SGM people have shown these items to be associated with mental and physical health outcomes. For example, outness, as used in this study, was found to be a robust predictor of physical health outcomes^17^. Experienced stigma items have also been associated with healthcare avoidance among GM people in previous research^16^.

### Future directions

Outness, or concealment, is also a complex construct. It may hold a protective role in some environments, yet also reflects a form of stress whose effects may manifest over longer periods of time^44,92^. Therefore, the relationships and effects between structural stigma and outness may not be evident until evaluated within the context of health outcomes longitudinally and, subsequently, warrant further study. Conceptual measurement concerns also remain salient for this outcome^92^, therefore continued evaluation of the relationship between structural stigma and outness as a form of minority stress is needed. The present study does not evaluate the role of protective factors, such as individual approaches to coping, community engagement, and pride in one’s GM identity. Examination of the relationship between structural stigma and protective factors in future analyses could address this consideration. Future investigations should consider sampling a priori a sufficient number of participants within each state to permit multilevel analyses to disentangle state and individual-level effects, and to estimate state-level effects reliably. Further, the advancement of this model should include the evaluation of structural stigma and minority stress on health or health behavior outcomes, such as mental health, physical health, quality of life, and healthcare engagement.

## Conclusions

Currently, available variables representing structural stigma often rely on solely the use of laws and other policies, despite the structural stigma’s conceptual definition that includes societal stigma. There are no variables currently developed for this purpose. The present study shows that structural stigma inclusive of societal stigma (State LGBT Business Climate Index) is associated with parts of the minority stress model (i.e., anticipated stigma, experienced stigma), in contrast to the more commonly used variable composed of laws and policies (i.e., State Policy Environment Tally) which was not associated with minority stress outcomes. However, one proxy variable (population density) was the sole structural stigma variable associated with outness, pointing to continued gaps in conceptual measurement. These findings support the use of variables for structural stigma that are inclusive of societal stigma as more appropriate approaches in research. However, future development of a measure expressly for assessment of structural stigma inclusive of societal stigma poses an opportunity to more closely understand minority stress experiences to identify priority areas for future intervention to address health equity barriers for GM people.

## Electronic supplementary material

Below is the link to the electronic supplementary material.

Supplementary Material 1

## Data Availability

The data that support the findings of this study are available from The PRIDE Study but restrictions apply to the availability of these data, which were used under license for the current study, and so are not publicly available. Data are however available from the corresponding authors upon reasonable request and with permission of The PRIDE Study.

## Abbreviations

GM: Gender minority
PRIDE: The Population Research in Identity and Disparities for Equality Study
SGM: Sexual and gender minority
