# Supplementary material for: Relationships between structural stigma, societal stigma, and minority stress among gender minority people
Source: Sci Rep. 2025 Jan 23;15:2996. doi: 10.1038/s41598-024-85013-8 (PMC11757992; doi:10.1038/s41598-024-85013-8)
Supplement: Supplementary file 1 — Supplementary Material 1 [file 41598_2024_85013_MOESM1_ESM.docx]

Supplemental Table 1. Unadjusted Model of Societal Stigma and Its Association with Minority Stress (N=2,094)

p<.05*, p<.01**, p<.001***

| Supplemental Table 1. Unstandardized estimates, standard errors, and standardized estimates for the structural equation model of minority stress and its association with structural stigma variables (unadjusted model; N=2,094). | | | | | | | | | | | | | | | | | | |
| --- | --- | --- | --- | --- | --- | --- | --- | --- | --- | --- | --- | --- | --- | --- | --- | --- | --- | --- |
| Outcome Variable | Google Trend | | | Movement Advancement Project | | | State LGBT+ Business Climate Index | | | Conservative Voting Behavior | | | US Region | | | Population Density | | |
|  | B | SE | β | B | SE | β | B | SE | β | B | SE | β | B | SE | β | B | SE | β |
| Experienced Stigma | 0.006 | 0.022 | 0.032 | 0.021 | 0.081 | 0.181 | **-0.023** | **0.025** | **-0.289** | **-2.082** | **0.086** | **-0.011** | 0.001 | 0.052 | 0.043 | 0.019 | 0.025 | -0.100 |
| Anticipated Stigma | 0.002 | 0.024 | 0.005 | 0.033 | 0.096 | 0.143 | **-0.057** | **0.106** | **-0.375** | -0.349 | 0.086 | -0.011 | 0.279 | 0.052 | 0.043 | **-0.944** | **0.025** | **-0.100** |
| Internalized Stigma | -0.003 | 0.052 | -0.028 | 0.006 | 0.094 | 0.091 | -0.002 | 0.135 | -0.041 | 0.554 | 0.070 | 0.060 | 0.069 | 0.044 | 0.037 | -0.103 | 0.026 | -0.038 |
| Outness | 0.012 | 0.025 | 0.043 | 0.006 | 0.100 | 0.032 | -0.005 | 0.166 | -0.045 | -1.721 | 0.077 | -0.070 | 0.109 | 0.038 | 0.022 | 0.244 | 0.028 | 0.034 |
| Bolded values indicate *p*<.05* | | | | | | | | | | | | | | | | | | |
